# A risk-based approach to measuring population micronutrient status from blood biomarker concentrations

**DOI:** 10.3389/fnut.2022.991707

**Published:** 2022-09-26

**Authors:** Santu Ghosh, Anura V. Kurpad, Harshpal S. Sachdev, Tinku Thomas

**Affiliations:** ^1^Department of Biostatistics, St. John's Medical College, St. John's National Academy of Health Sciences, Bangalore, India; ^2^Department of Physiology, St. John's Medical College, St. John's National Academy of Health Sciences, Bangalore, India; ^3^Department of Pediatrics and Clinical Epidemiology, Sitaram Bhartia Institute of Science and Research, New Delhi, India

**Keywords:** nutrient biomarkers, risk of deficiency, deficiency cut-off, population prevalence, children

## Abstract

**Background:**

Nutrient biomarkers and their definitive cut-offs are used to classify individuals as nutrient-deficient or sufficient. This determinism does not consider any uncertainty, and a probability approach, using biomarker distributions, is then preferable to define the risk of nutrition deficiency when in populations.

**Method:**

Healthy 1–19-year-old children and adolescents were selected from the Comprehensive National Nutrition Survey (CNNS), to obtain probability distributions of their retinol, zinc and vitamin B_12_, along with erythrocyte folate. Model-based estimates of location, scale and shape parameters of these distributions were obtained across ages. Subsequently, in the entire sample of 1–19 year old children of CNNS, the population risk of deficiency (PRD) which is average risk of deficiency in individuals in the population was computed, which is “of concern” when >50%. When individual risk of deficiency is >97.5% it is called “severe risk of deficiency” (SRD).

**Results:**

In the entire CNNS sample, the PRD of concern was low for serum retinol (3.6–8.2%), zinc (0–5.5%), and SRD of vitamin B_12_ and erythrocyte folate were 2.3–7.2% and 4.2–9.7%, respectively, across age and sex groups.

**Conclusion:**

This proposed method assesses the adequacy of nutrient exposures without relying on pre-defined deterministic biomarker cut-offs to define micronutrient deficiency and avoids errors in exposure assessment.

## Introduction

The access to sufficient food, of adequate quality, to maintain normal body composition and function throughout the life-cycle, is considered fundamental to maintaining good health (1). The objective measurement of “sufficient food or nutrients” could be made through the measurement of specific nutrient status in the body and is expressed in terms of its adequacy or deficiency. As nutrient intakes have improved temporally, this assessment has moved from the evaluation of profound nutrient deficiency by frank clinical signs, to the evaluation of more subtle deficiency from blood or urine nutrient (biomarker) concentrations.

The Biomarkers of Nutrition for Development (BOND) consortium began the process of evaluating the application of specific biomarkers of micronutrients for the definition of micronutrient malnutrition (2). In the subclinical state, without any obvious signs or symptoms of deficiency, the best status indicator would be a decline in functionality. However, owing to a lack of well-defined function indicators in many instances, or a high variability in these measurements, a deficient (or replete) status was defined by a diagnostic serum/urine biomarker-based cut-off. This in turn was based on the general practice of defining the distribution of the biomarker in a well-defined healthy population, and then identifying the diagnostic cut-off as the value corresponding to the 2.5–5th percentile value of the distribution. Even so, while health can be defined based on a variety of socio-economic, anthropometric and other biochemical markers (3, 4), the complete definition of health as a state of physical, mental and social wellbeing, and not merely the absence of disease or infirmity (5), is also contextual, and easier said than done. For example, populations can adapt to lower nutrient intakes while maintaining good health, without physiological cost.

The fundamental problem with the cut-off approach is the deterministic or definitive diagnosis of nutrient deficiency, such that values below the cut-off are deemed deficient with certainty (probability = 1), when it is known that there will be some level of uncertainty in the cut-off itself in any individual, due to the definition of health employed and the factors referred to above. This uncertainty is ignored. Moreover, the cut-off will artificially differentiate individuals, with similar health characteristics, but with biomarker values lying in the neighborhood of the cut-off, into deficient or replete. Another problem is the possibility that the distribution of biomarkers of a healthy population may vary from one geographical or economic area to another, due to costless adaptations to different habitual dietary nutrient intakes, or due to unique variations in different populations. Different distributions mean different diagnostic cut-offs, making a single global cut-off value unlikely (3).

The risk-based, probabilistic approach for estimating the inadequacy of nutrient intake in a population can be used here. Thus, the individual, as well as population, risk of nutrient deficiency can be evaluated by comparing the measured biomarker against the distribution of the biomarker in a context-specific healthy population. In this paper, a probability approach to define the risk of nutrition deficiency is described, by defining the distribution of select micronutrient blood biomarkers in healthy Indian children aged between 1 and 19 years.

## Methods

The data for this analysis were obtained from the Comprehensive National Nutrition Survey (CNNS) (6) a cross-sectional, nationally representative survey of Indian children and adolescents, which was conducted in 30 states of India between 2016 and 2018, under the aegis of the Ministry of Health and Family Welfare, Government of India, in collaboration with United Nations International Children's Emergency Fund (UNICEF), India and the Population Council, Delhi, India. The survey design and sampling methodology are already published elsewhere (6–8). Briefly, a multi-stage, population proportional to size cluster sampling was done to enroll preschool (1–4 years), school age (5–9 years) children, and adolescents (10–19 years), to adequately represent the national, state, male-female, and urban-rural population. For blood sampling, 50% of all the children who completed anthropometry were contacted through systematic random sampling. Children/adolescents with physical deformity, cognitive disabilities, chronic illness, acute febrile/infectious illness, acute injury, ongoing fever and pregnancy were excluded (6).

The Population Council's International Review Board (New York, USA) and the Ethics Committee of the Post Graduate Institute of Medical Education and Research (Chandigarh, India) gave ethical approval (6). Written consent from the parent/caregiver for children <10 years, consent of parent/caregiver as well as assent for adolescents between 11 and 17 years, and written consent of adolescents >17 years were obtained after due description of study details in local languages.

Household socioeconomic and demographic characteristics, and information on history of morbidity in the preceding 2 weeks, along with anthropometric data of one child/adolescent per age group, were collected from each household; these methods are detailed elsewhere (6). The Wealth Index, based on possession of common household items and facilities, was computed as described in the National Family Health Survey-4 (9). Access to facilities like drinking water, hand washing and sanitation was categorized based on the World Health organization (WHO)/UNICEF Joint Monitoring Program guidelines (10). Age- and sex-standardized height-for-age (HAZ), weight-for-age (WAZ) and body mass index (BMI) -for-age (BAZ) Z-scores were calculated using the WHO Growth Reference (11, 12). Blood sample collection procedure and biomarker analysis methods have been detailed elsewhere (6, 13), but briefly, venous blood samples with information on fasting status were collected in trace element free tubes (Greiner Bio One, India) to allow the measurement of all biomarkers of interest. The tubes were transported to the nearest laboratory on ice and serum/red blood cells separated within 6 h from the time of sample collection. The detailed quality assurance of biological samples were described in CNNS Report (6).

A sample of apparently healthy children was selected out of the CNNS dataset, relying on available indicators such as sociodemographic characteristics and blood biomarkers (Supplementary Figure 1). We first excluded participants from the lower three quintiles of socioeconomic index assuming upper two quintiles are likely to have the best environmental conditions to be healthy. Next, households with unimproved drinking water, and poor to moderate level of sanitation were excluded. We also excluded those children with WAZ, HAZ, WHZ, and BAZ values <-2SD according to the WHO standards; anemic children (6) and those with hypoalbuminemia (serum albumin <3.5 g/dL); those with BMI above 97.5th percentile of the standard (for children >5 y), with elevated HbA1c (>5.9 g/dL) and history of smoking. Other exclusion criteria included children with any infection, such as fever or diarrhea in the 2 weeks prior to interview, or those with serum C-reactive protein (CRP) >5 mg/L. The association of all household and individual characteristics that were considered as inclusion and exclusion criteria for the definition of health, with biomarkers, was examined prior to the extraction of the healthy sample. All factors were found to be associated significantly (or close to significantly) with biomarkers in at least any one of the age groups of children (Supplementary Figures 2–5). Finally, to avoid over-dispersion due to unobserved systematic variability, we excluded data outside the lower 5th and upper 5th percentiles of respective biomarkers at the time of analysis. After these exclusions, data on serum retinol (SR), zinc (SZ), vitamin B_12_ (Vit B_12_) and erythrocyte folate (folate) were abstracted. The assays used for the analysis of the biomarkers are as follows- for SR: HPLC Reverse phase chromatography; for SZ: Flame Atomic Absorption spectrometry with D2 correction; for erythrocyte folate: Competitive immunoassay using direct chemiluminescence; for Vit B_12_: Competitive immunoassay using direct chemiluminescence (6). The challenge of developing a standard distribution of biomarkers is having sufficient data of their measurement in a healthy population. In the selection of sub-sample for SZ, we ignored fasting status because (i) there were insufficient representation of fasting blood for some age and sex groups, specially under 5 years children and (ii) distribution was observed to be quite similar across age and sex group by fasting status (14). We performed a simulation exercise (Supplementary Figure 6) to demonstrate that a healthy population which is expected to have the best distribution of the biomarker can be identified as a subsample of a nationally representative survey.

Next, to develop a standard distribution of each biomarker in these healthy children, we preferentially used a parametric approach wherever a normal or lognormal probability distribution of biomarker could be suitably fitted by maximum likelihood estimation (MLE). We chose a Z-score approach by the LMS method (15) for other biomarkers, for simplicity. However, to smoothen the distribution of nutritional biomarkers over years of age, and to eliminate any unusual fluctuation due to varying numbers of children at each year of age, we applied the generalized additive model for location scale and shape (GAMLSS) with Box-Cox-Cole-Green transformation to all biomarkers. The MLE method suggested a Normal probability distribution for SZ concentration, a Log Normal probability distribution for SR concentrations, and a Weibull or Gamma probability distribution for Vit B_12_ and folate concentrations. A GAMLSS model was applied on SZ and log (SR), assuming a symmetric shape across age. However, for Vit B_12_ and folate concentrations, we relaxed the symmetric assumption and allowed the algorithm to estimate the parameters of location, scale and shape across ages. The specifications of the sub-component of GAMLSS model such as mean model, variance model and the model corresponding to shape of the distribution were based on *p*-values of the relevant components of the distribution estimated for each of the markers. The distributions of the standards were aggregated for four age groups (1–4 y, 5–11 y, 12–14 y, 15–19 y), stratified by sex.

We suggest using the term “risk of deficiency” as an alternative metric to the more deterministic “deficiency” for assessing micronutrient status. This metric is based on the serum/blood biomarker concentration, by comparing this with a standard distribution of the same biomarker concentration derived in healthy age- and sex-matched individuals. Assume Y is a random variable which denotes the serum level of the marker in a healthy population for a given age and sex, with a probability distribution *D*(*y*; μ, σ, λ), where, μ, σ and λ are location, scale and shape parameters respectively. If the concentration of the selected biomarker of an individual with same age and sex is x, the risk of micronutrient deficiency is defined by following equation.

(1) $$\begin{array}{l} r\left(x\right)=Prob\left(Y>x\right) \end{array}$$

For example, if *Y* ~ *Norm*(μ, σ^2^) then the risk of deficiency can be derived by

(2) $$\begin{array}{l} r\left(x\right)=Prob\left(Y>x\right)=1-Prob\left(Y\leq x\right)=1-\Phi \left(\frac{x-\mu }{\sigma }\right) \end{array}$$

The MS Excel (Microsoft Office 365) macro “1 − *NORM*.*DIST* (*x*, μ, σ, *TRUE*)” can be used to derive this value for an individual.

In case *Y* ~ *LogNormal*(μ, σ^2^), then the risk can be derived by

(3) $$\begin{array}{l} r\left(x\right)=Prob\left(Y>x\right)=1-\Phi \left(\frac{\log\left(x\right)-\mu }{\sigma }\right) \end{array}$$

The corresponding MS Excel macro will be “1 − *NORM*.*DIST*(*LOG*(*x*), μ, σ, *TRUE*)”.

However, for a non-parametric distribution with the standard derived by LMS method (14), the derivation would be somewhat complicated but is still manageable in MS Excel too. For a given {μ, σ&λ} Z-score by LMS method can be derived by the following equations (14).

(4) $$\begin{array}{l} Z\left(x\right)=\frac{{\left(\frac{x}{\mu }\right)}^{\lambda }-1}{\lambda \sigma }\;for\;\lambda \neq 0 \end{array}$$

(5) $$\begin{array}{l} Z\left(x\right)=\frac{\log\left(\frac{x}{\mu }\right)}{\sigma }\;for\;\lambda =0 \end{array}$$

The risk of deficiency in this case can be calculated by

(6) $$\begin{array}{l} r\left(x\right)=Prob\left(Y>x\right)=Prob\left(Z\left(Y\right)>Z\left(x\right)\right)=1-\Phi \left(Z\left(x\right)\right) \end{array}$$

Therefore, Z(x) should be derived using equation (4) if estimated λ ≠ 0, otherwise equation (5). Then the macro “1 − *NORM*.*DIST*(*Z*(*x*), 0, 1, *TRUE*)” should derive the desired risk using MS Excel.

The population risk of deficiency (PRD) for any micronutrient can be measured as the average risk of deficiency of the individuals. By probability theory, the risk of micronutrient deficiency of an individual will be “of concern” only when the risk is >50%, that is, when the blood/serum concentration of a micronutrient biomarker of an individual is below the median of the standard distribution of the biomarker in a healthy matched population. Then, the average risk of micronutrient deficiency of a population at 50% can be considered as the threshold of true population risk of micronutrient deficiency.

Often in practice, a sufficiently representative random sample of a population is unavailable for assessment of population risk. Here, a completely parametric approach with prior knowledge on the shape of the distribution of biomarker concentration in a population will increase the chance of a correct assessment of population risk of deficiency. If we know that the population distribution of biomarker concentration is symmetric, like SZ for example, or positively skewed, like SR for example, we can assume a normal or lognormal probability distribution of the marker, respectively, for the given population. The mean and standard deviation (SD) for a normal distribution and log-scale mean and SD for a lognormal distribution are the unbiased estimators of location and scale parameters, respectively. If X is a random variable that denotes the serum level of a biomarker in a given population, with known probability distribution *f*_*p*_(*x*; μ, σ) ; μ & σ are location and scale parameters, respectively. The PRD for any micronutrient can be defined by

(7) $$\begin{array}{l} Prob\left(Y>X\right)=\int_{a}^{b}r\left(x\right)f_{p}\left(x\right)dx=E_{X}\left\{r\left(x\right)\right\}\approx \frac{1}{n}\overset{n}{\underset{i=1}{\sum }}r\left(x_{i}\right) \end{array}$$

with the assumption of independence between X and Y and a large simulated random sample from *f*_*p*_(*x*; μ, σ) ; *a* < *x* < *b*.

$r\left(x\right)=Prob\left(Y>x\right)=\int_{x}^{\infty }g\left(y\right)dy$; *g*(*y*) is the parametric distribution of the standard.

However, for a standard derived by LMS method, one can derive


$$\begin{array}{l} r\left(x\right)=Prob\left\{Z\left(Y\right)>Z\left(x\right)\right\}=1-\Phi \left(Z\left(x\right)\right); \end{array}$$

where Φ(·) is the cumulative distribution function of standard normal probability distribution.

If an individual risk of micronutrient deficiency is >97.5%, this is termed “severe risk of deficiency” (SRD), meaning that the biomarker concentration for that individual is lower than the 2.5th percentile of the standard distribution of the biomarker. This is equivalent to the existing single cut-off metric for blood/serum micronutrient biomarkers, to define micronutrient deficiency.

The proposed standards for serum concentrations for SR, SZ, B_12_, and folate, derived from the “healthy” sub-sample of CNNS children, were then applied to the entire CNNS data to estimate the PRD and prevalence of SRD (or the current understanding of prevalence of micronutrient deficiency) at the national level. The population distribution of SR concentration was approximated by a log normal distribution and SZC by a normal probability distribution, after correcting for inflammation, based on the CRP (16). Equation −7 was applied to estimate population risk of deficiency for these three micronutrients. The data were analyzed using statistical software R version 4.1.0 (R Core Team, 2021, Vienna, Austria).

## Results

The CNNS survey published biomarker data of 49,486 children, aged 1–19 years. After applying the exclusion criteria reported in the methods section to derive a “healthy” sample of children, a varying number of valid micronutrient biomarker measurements were available. These numbers were, SR (9506); SZ (9966); Vit B_12_ (9699); and folate (11,220). The detailed age and sex specific frequency distributions are reported in Supplementary Table 1.

**Figure 1:**
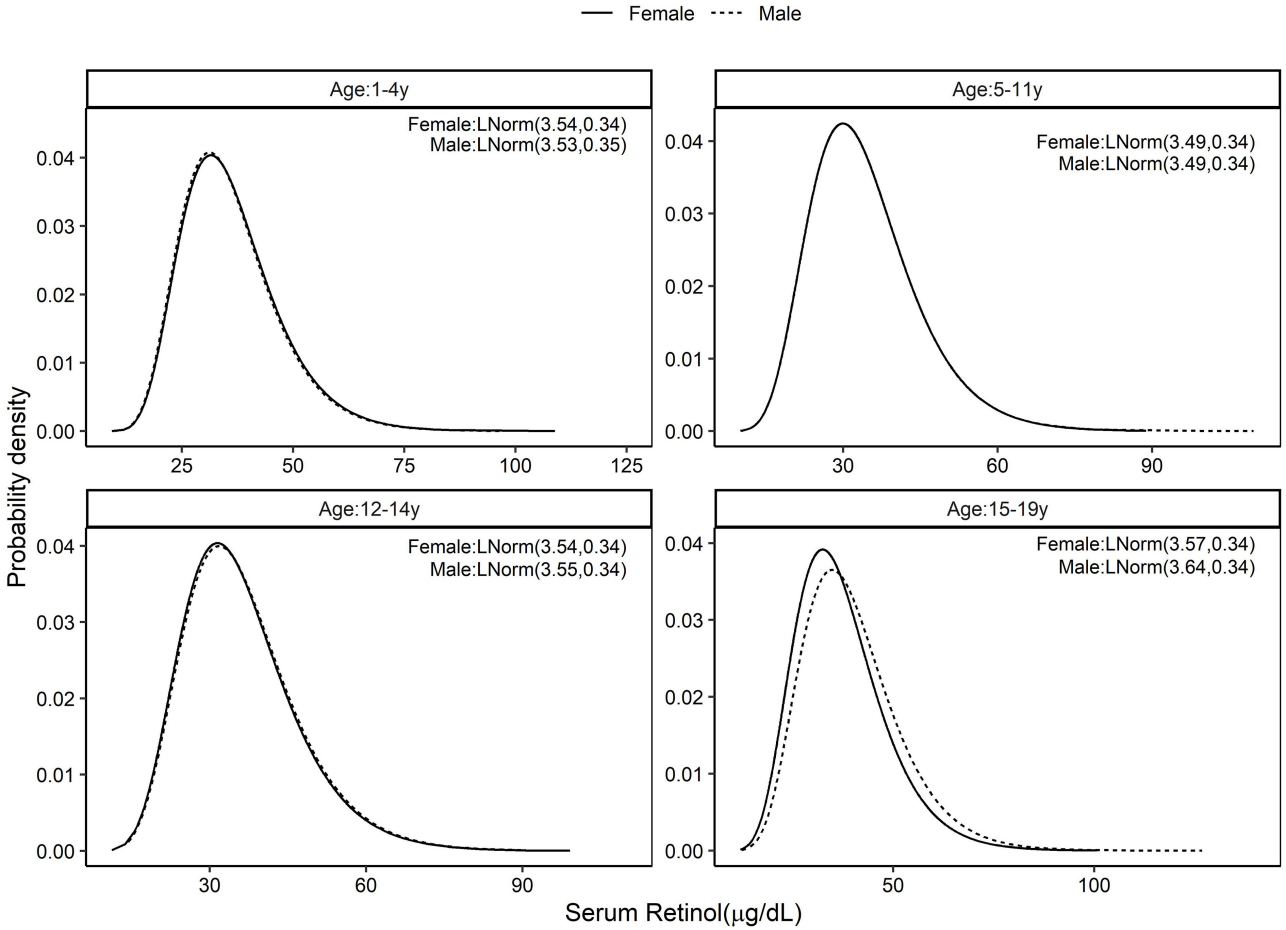
Standard distribution of serum retinol (μ*g*/*dL*) concentrations among healthy Indian children aged 1–4, 5–11, 12–14, and 15–19 years; Solid line represents Female children and dotted line represents Male children. Values given in parenthesis are location and scale parameters of the lognormal distribution of serum retinol in μ*g*/*dL*.

**Figure 2:**
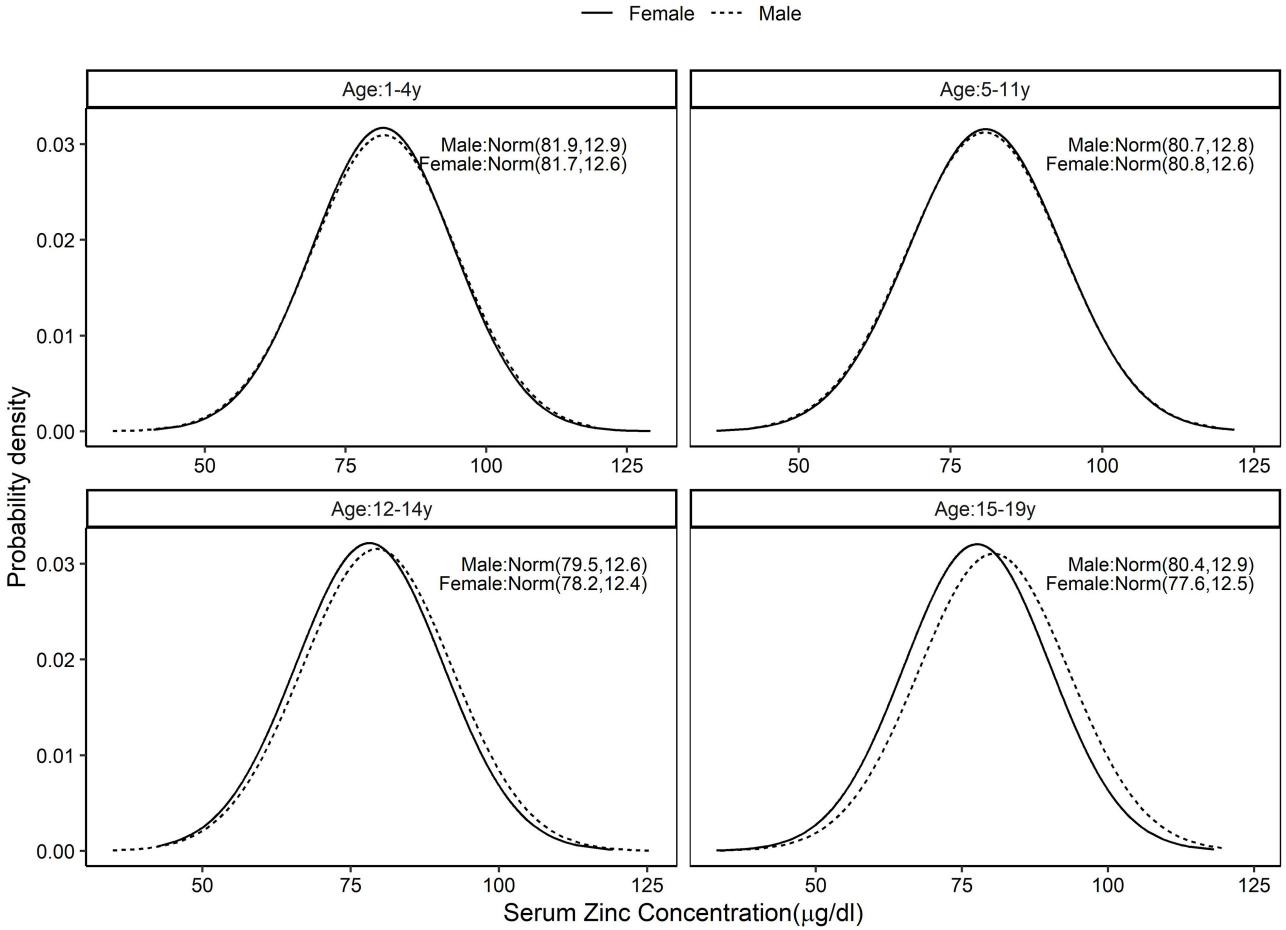
Standard distribution of serum zinc (μ*g*/*dL*) concentrations among healthy Indian children aged 1–4, 5–11, 12–14, and 15–19 years; Solid line represents Female children and dotted line represents Male children. Values given in parenthesis are mean and SD of normally distributed serum zinc in μ*g*/*dL*.

The standard distribution of SR (μg/dL) was estimated as a log normal probability distribution with location and scale parameters given in Table 1. Then, using the entire CNNS dataset, the national PRD for SR varied from 53.6 to 58.2% across different age and sex specific groups. Effectively, this would mean a nutritional concern, or the risk of a true population deficiency, in 3.6–8.2% of the population. The prevalence of SRD for SR, or equivalently, prevalence of vitamin A deficiency as currently understood, varied from 9.1 to 10.4% across the groups (Figure 1; Table 1). The standard distribution for SZ (μg/dL) was also estimated as a normal probability distribution with location and scale parameters as in Table 1. The national estimate of PRD for SZ varied from 48.3 to 55.5%, or as a nutritional concern, from 0 to 5.5% across different age and sex specific groups, while the prevalence of SRD of SZ varied from 5 to 8% across the groups (Figure 2; Table 1).

**Table 1:** The estimated standard of serum retinol and zinc across age & Sex with national estimate of population risk of deficiency (PRD) and prevalence of severe risk of deficiency (SRD).

**Age and sex**	**Standard**		**National**	
	**distribution**		**estimate**	
	**Location**	**Scale**	**PRD (%)**	**Prev. SRD (%)**
**Serum retinol (μg/dL): log-normal distribution**				
Male: 1–4 y	3.53	0.35	56.6	9.3
Female: 1–4 y	3.54	0.34	56.6	10.2
Male: 5–11 y	3.49	0.34	57.1	10.1
Female: 5–11 y	3.49	0.34	58.2	10.4
Male: 12–14 y	3.55	0.34	56.6	12.3
Female: 12–14 y	3.54	0.34	53.6	10.1
Male: 15–19 y	3.64	0.34	56.5	9.9
Female: 15–19 y	3.57	0.34	54.7	9.1
**Serum zinc (μg/dL): normal distribution**				
Male: 1–4 y	81.9	12.9	53.1	6.4
Female: 1–4 y	81.7	12.6	55.5	8.0
Male: 5–11 y	80.7	12.8	49.6	5.3
Female: 5–11 y	80.8	12.6	50.4	5.8
Male: 12–14 y	79.5	12.6	52.4	5.6
Female: 12–14 y	78.2	12.4	50.8	5.4
Male: 15–19 y	80.4	12.9	48.3	5.0
Female: 15–19 y	77.6	12.5	50.0	6.2

The folate distribution (ng/ml) was also estimated by LMS method (Table 2). The national estimate of PRD for folate varied from 42 to 56.8% across different age and sex specific groups (low nutritional concern), while the prevalence of SRD for folate varied from 4.2 to 9.7% across different age and sex specific groups (Supplementary Figure 7; Table 2). Similarly, the standard distribution for Vit B_12_ was also estimated by LMS method (Table 2). The national estimate of PRD for Vit B_12_ varied from 51.7 to 62.4% across different age and sex specific groups (low nutritional concern), while the prevalence of SRD for Vit B_12_ varied from 2.3 to 7.2% across the groups (Supplementary Figure 8; Table 2). The 2.5th percentile values of all the biomarkers corresponding to SRD and the equivalent to existing cut-off for the biomarkers are provided in the Supplementary Table 1.

**Table 2:** Standard distribution for erythrocyte folate, and serum vitamin B_12_ concentrations derived by the LMS method across age and sex with national estimate of population risk of deficiency (PRD) and prevalence of severe risk of deficiency (SRD).

**Age and sex**	**Standard distribution**			**National estimate**	
	**μ**	**σ**	**λ**	**PRD (%)**	**SRD (%)**
**Erythrocyte folate (ng/mL)**					
Male: 1–4 y	316.77	0.51	0.43	56.1	8.2
Female: 1–4 y	312.65	0.51	0.42	56.8	9.7
Male: 5–11 y	254.58	0.55	0.43	51.6	5.4
Female: 5–11 y	242.22	0.57	0.42	50.3	4.7
Male: 12–14 y	214.79	0.60	0.43	51.7	4.4
Female: 12–14 y	206.45	0.61	0.42	47.9	3.4
Male: 15–19 y	192.91	0.60	0.43	51.5	4.6
Female: 15–19 y	204.79	0.59	0.42	42.0	4.2
**Vitamin B**_**12**_ **(pg/mL)**					
Male: 1–4 y	367.04	0.34	−0.16	62.4	5.0
Female: 1–4 y	375.25	0.34	−0.07	62.7	7.2
Male: 5–11 y	321.79	0.32	−0.34	57.8	5.0
Female: 5–11 y	328.79	0.32	−0.16	59.6	6.4
Male: 12–14 y	262.99	0.30	−0.50	57.7	3.5
Female: 12–14 y	278.31	0.31	−0.31	57.3	6.4
Male: 15–19 y	232.88	0.30	−0.63	51.7	2.3
Female: 15–19 y	257.20	0.31	−0.25	52.3	3.7

## Discussion

In this report, we describe a probability approach for defining the risk of deficiency of certain micronutrients in healthy Indian children aged between 1 and 19 years, using the standard distribution of their blood biomarkers. A collection of criteria were used to identify a healthy subset of the CNNS survey data to represent a standard healthy population of Indian children. The PRD defined for these nutrients were used to identify the proportion of population which would be below the median of the standard distribution. This percentage was close to, or less than, the threshold (50%) for SZ and about 10% for all other nutrients in the entire nationally representative sample of CNNS. These deficiency estimates are lower than what is currently thought based on global biomarker cut-offs. However, given that global cut-offs are based on available high-income country studies, while the present, being contextual and based on a healthy local population, are likely to be more appropriate and precise. This was explicitly stated by a WHO committee tasked with identifying indicators for assessing vitamin A deficiency (3). In that report, they stated, “(The) interpretation of vitamin A deficiency status depends on the availability of reference data…from elite groups within the population itself.” It is known that populations can adapt to lower nutrient intakes up to a point; perhaps the best example comes from the classic studies of Chittenden over a century ago (17), who showed that university athletes and army recruits could maintain excellent fitness and muscularity on what was then (and perhaps even now) considered a low protein diet, in comparison to the high standard for protein intake at that time.

The main purpose of nutritional biomarkers can either be the validation of dietary intake instruments, or their functioning as surrogate indicators of dietary intake or exposure, or even their functioning as an integrated measure of nutritional status in the population for a particular nutrient. However, a major problem that relates to biomarker based assessment is the misclassification of exposure (18). The classification of exposure is classically based on a biomarker cut-off. Modifying the Institute of Medicine (IOM) definition of a biomarker cut-off (19), the nutrient biomarker is expected to demarcate the presence or absence of a nutritional exposure; but the challenge is in reliably distinguishing the exposure based on a cut-off from a continuum of biomarker values. Misclassification can lead to underestimation or to overestimation of the impact of exposure and can even have the wrong sign (deficiency or sufficiency). Thus, the precision of the classification of deficiency or repleted-ness greatly depends on the reliability and validity of the biomarker measurement itself. However, the risk of deficiency approach, as proposed here, overcomes the mis-classification problem of the biomarker cut-off-based approach.

Another problem with a biomarker cut-off is in its quantitative identification from the distribution in a carefully defined healthy population, by identifying a certain percentile of the distribution to be the cut-off for deficiency, which can then classify the exposure (intake) to the nutrient. For example, the cut-off of anemia corresponds to the 4.95th percentile of the distribution of hemoglobin in a healthy women (20). This is in stark contrast to defining the biomarker cut-off based on functional outcomes or exposure-outcome associations. This is worth noting, since underlying biological variations in individual assessments, and factors related to laboratory methods and assays, can introduce variations in the cut-off that is eventually chosen to represent nutrient deficiency (21). The actual distribution of the nutrient biomarker in the general population represents a continuum from deficiency to excess, but it is common practice to arbitrarily break down the biomarker to reflect 3 categories of dietary intake: inadequate, adequate, and excess (21). The proposed approach of identifying the risk of deficiency based on distribution of the biomarker provides a continuum of exposure, retaining the original nature of the measurement as a continuous variable, rather than dichotomizing or categorizing it.

The risk-based approach to characterize nutrient deficiency using a biomarker is analogous to the approach used in identifying inadequacy of dietary nutrient intake, where the estimation of a risk or probability of inadequacy at the population level has been recommended by bodies like the Institue of medicine (19). The measurement of dietary inadequacy is based on the estimated average requirement of nutrients in a population, based on the daily physiological losses of the nutrient and the efficiency of replacing these losses through the diet. Similarly, the risk of nutrient deficiency proposed in this paper presents the population, rather than individual, level risk of deficiency. This was performed for serum SR, SZ, Vit B_12_ and erythrocyte folate in male and female Indian children aged 1–19 years based on the distribution of the respective biomarker in a carefully selected healthy sub-sample of children to mimic a reference population for India. Unfortunately, robust national data on the dietary nutrient intake of children does not exist. Therefore, a validation of our estimates against risk of dietary inadequacy is not possible.

The strength of this study is that the distribution of biomarkers was defined using a well-characterized subsample of healthy children aged 1–19 y and therefore this study serves as a demonstration of the method proposed. While there are several advantages of a risk-based approach, the biggest challenge still lies in equating the risk from the distribution of biomarker to a functional or disease outcome. Further studies are required in this matter. For example, it will be very useful to understand how the distribution of Vit B_12_ concentrations corresponds to the distribution of Vit B_12_ related function, which is to act as a co-factor in the conversion of methylmalonyl CoA into downstream succinyl CoA and uncertainty exists on the best way to define Vit B_12_ deficiency or equally, how the distribution of SR correlates with the distribution of adverse health outcomes such as Bitot's spot or night blindness. Another challenge is in the identification of the correct probability distribution and its parameters for the biomarker. In the set of nutrients considered in this study, the SZ concentrations followed a normal distribution, while SR concentration followed a log normal distribution, and Vit B_12_ and folate followed a Weibull or Gamma probability distribution. A further challenge is that many low- and middle-income countries (LMIC) may not have contemporary surveys that provide nutrition biomarker distributions in their healthy populations. However, it must be argued that the interests of nutrition policy-making in LMIC are best served by their own local biomarker surveys, to provide the much-needed evidence base to define locally relevant cut-offs.

In conclusion, the proposed method of risk, or probability of deficiency, offers a method of assessing nutrient-based exposures without relying on cut-offs of deficiency which are prone to errors in assessment of exposure.

## Data availability statement

The datasets presented in this article are not readily available because they are the property of the Ministry of Health and Family Welfare, Govt. of India. Requests to access the datasets should be directed to Ministry of Health and Family Welfare, Govt. of India.

## Ethics statement

The studies involving human participants were reviewed and approved by the Population Council's International Review Board (New York, USA) Ethics Committee of the Post Graduate Institute of Medical Education and Research (Chandigarh, India). Written informed consent to participate in this study was provided by the participants' legal guardian/next of kin.

## Author contributions

SG and TT conceptualized the study and drafted the manuscript. SG performed the modeling and analysis. AK and HS provided critical review while drafting the manuscript. All authors read and approved the manuscript.

## Funding

AK, HS, and TT are recipients of the Wellcome Trust/Department of Biotechnology India Alliance Clinical/Public Health Research Center Grant # IA/CRC/19/1/610006.

## Conflict of interest

Author HS is a member of the World Health Organization Nutrition Guidance Expert Advisory Group (NUGAG) Subgroup on Diet and Health and a member of Expert Groups of the Ministry of Health and Family Welfare on Nutrition and Child Health. The remaining authors declare that the research was conducted in the absence of any commercial or financial relationships that could be construed as a potential conflict of interest.

## Publisher's note

All claims expressed in this article are solely those of the authors and do not necessarily represent those of their affiliated organizations, or those of the publisher, the editors and the reviewers. Any product that may be evaluated in this article, or claim that may be made by its manufacturer, is not guaranteed or endorsed by the publisher.

## Supplementary material

Supplementary Figure 1 Flowchart of sequential participant exclusion for the analytical samples. WAZ, WHZ and BAZ are abbreviations for Weight-for-age, weight-for-height and BMI-for-age Z scores based on WHO growth standards.

Supplementary Figure 2 The direction of association of serum retinol and factors used for extraction of healthy subsample of Comprehensive National Nutrition Survey across three age groups (1–4 y, 5–9 y, 10–19 y). HAZ, WAZ, WHZ and BAZ are abbreviations for height-for-age, weight-for-age, weight-for-height and BMI-for-age Z scores based on WHO growth standards.

Supplementary Figure 3 The direction of association between serum zinc concentration and factors used for extraction of healthy subsample of Comprehensive National Nutrition Survey across three age groups (1–4 y, 5–9 y, 10–19 y). HAZ, WAZ, WHZ and BAZ are abbreviations for height-for-age, weight-for-age, weight-for-height and BMI-for-age Z scores based on WHO growth standards.

Supplementary Figure 4 The direction of association between serum erythrocyte folate and factors used for extraction of healthy subsample of Comprehensive National Nutrition Survey across three age groups (1–4 y, 5–9 y, 10–19 y). HAZ, WAZ, WHZ and BAZ are abbreviations for height-for-age, weight-for-age, weight-for-height and BMI-for-age Z scores based on WHO growth standards.

Supplementary Figure 5 The direction of association between serum vitamin B_12_ and factors used for extraction of healthy subsample of Comprehensive National Nutrition Survey across three age groups (1–4 y, 5–9 y, 10–19 y). HAZ, WAZ, WHZ and BAZ are abbreviations for height-for-age, weight-for-age, weight-for-height and BMI-for-age Z scores based on WHO growth standards.

Supplementary Figure 6 **(A)** Three randomly simulated sample data with three different ranges for an arbitrary variable. **(B,C)** Sampling distribution of mean and standard deviation of the random subset of upper 1/3 range of the arbitrary variable.

Supplementary Figure 7 Standard distribution of erythrocyte folate concentrations among healthy Indian children (2.5, 25, 50,75, and 97.5th centiles); Y-axis represents the erythrocyte folate concentration (*ng/mL*) and X-axis age of children in years. Lowest line corresponds to 2.5th percentile and the highest line corresponds to the 97.5th percentile.

Supplementary Figure 8 Standard distribution of vitamin B_12_ concentrations among healthy Indian children (2.5, 25, 50,75, and 97.5th centiles); Y-axis represents serum vitamin B_12_ concentration (*pg/mL*) and X-axis age of children in years. Lowest line corresponds to 2.5th percentile and the highest line corresponds to the 97.5th percentile.
