# Supplementary material for: A risk-based approach to measuring population micronutrient status from blood biomarker concentrations
Source: Front Nutr. 2022 Sep 26;9:991707. doi: 10.3389/fnut.2022.991707 (PMC9548994; doi:10.3389/fnut.2022.991707)
Supplement: Supplementary Figure 1 — Flowchart of sequential participant exclusion for the analytical samples. WAZ, WHZ and BAZ are abbreviations for Weight-for-age, weight-for-height and BMI-for-age Z scores based on WHO growth standards. [file Data_Sheet_1.zip › Figure S1.docx]

Online Supplemental Figure 1: Flowchart of sequential participant exclusion for the analytical samples.

67507 participants approached for blood collection

18021 refused to participate

49486 blood samples collected

Excluded: Smoking, High CRP, Diarrhea, Fever,

High Hb1AC, Stunted, WAZ<-2, WHZ<-2, BAZ(>5y) < -2 & >2, Albumin<3.5g/dL and Lower 3 wealth quintiles, households with unimproved drinking water, poor to moderate level of sanitation

Vitamin B_12_

: 9699

Erythrocyte Folate

:11220

Serum Zinc

: 9966

Serum Retinol

: 9506
