# Supplementary material for: A risk-based approach to measuring population micronutrient status from blood biomarker concentrations
Source: Front Nutr. 2022 Sep 26;9:991707. doi: 10.3389/fnut.2022.991707 (PMC9548994; doi:10.3389/fnut.2022.991707)

Online Supplemental Figure 6: A depicts the three randomly simulated sample data with three different ranges of an arbitrary variable. B and C depict the sampling distribution of mean and standard deviation of the random subset of upper 1/3 range of the arbitrary variable.


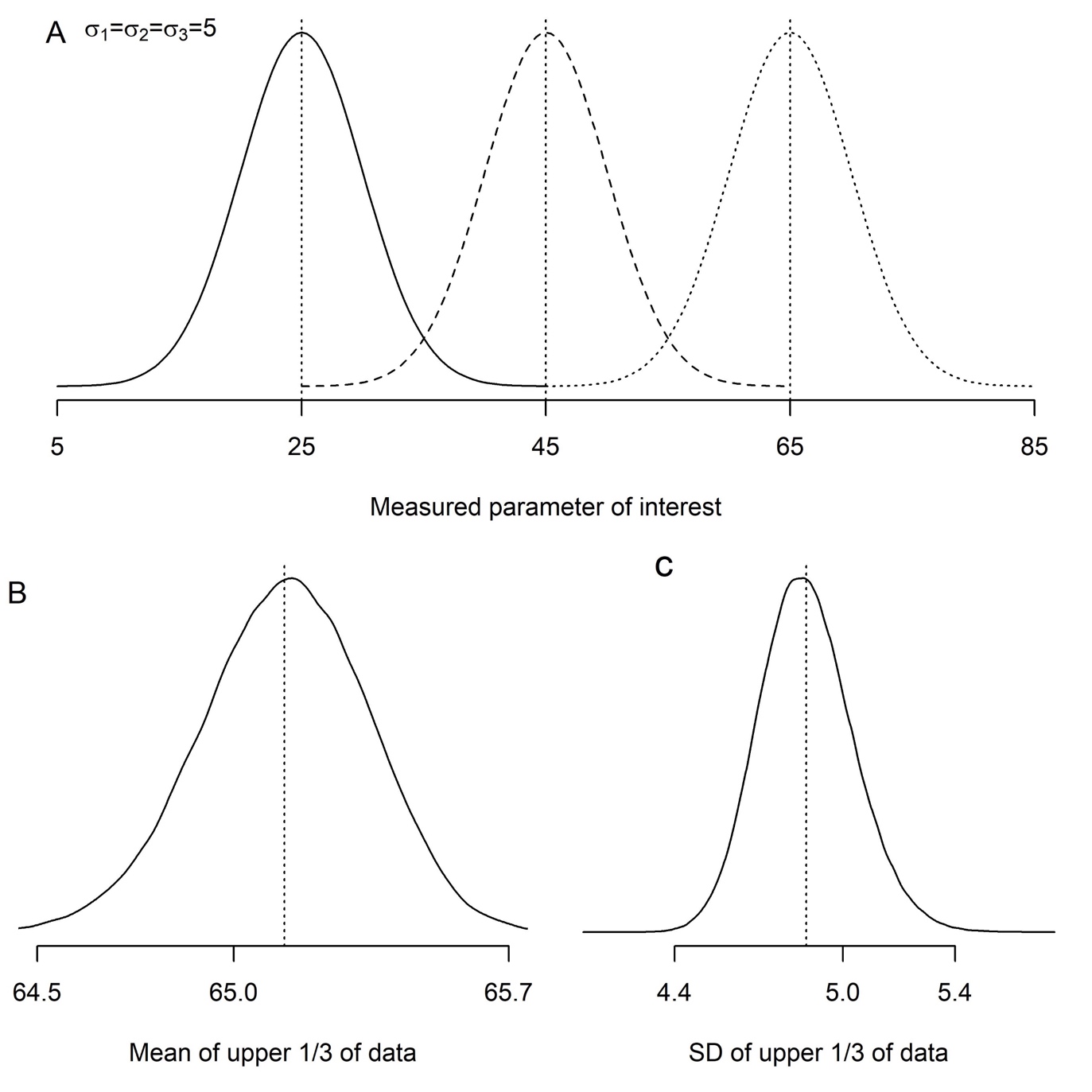

Supplement: Supplementary Figure 1 — Flowchart of sequential participant exclusion for the analytical samples. WAZ, WHZ and BAZ are abbreviations for Weight-for-age, weight-for-height and BMI-for-age Z scores based on WHO growth standards. [file Data_Sheet_1.zip › Figure S6.docx]
