# Supplementary material for: A risk-based approach to measuring population micronutrient status from blood biomarker concentrations
Source: Front Nutr. 2022 Sep 26;9:991707. doi: 10.3389/fnut.2022.991707 (PMC9548994; doi:10.3389/fnut.2022.991707)
Supplement: Supplementary Figure 1 — Flowchart of sequential participant exclusion for the analytical samples. WAZ, WHZ and BAZ are abbreviations for Weight-for-age, weight-for-height and BMI-for-age Z scores based on WHO growth standards. [file Data_Sheet_1.zip › Table S1.docx]

**Supplemental Table-1:** Values corresponding to 2.5^th^ percentiles derived from each standard distribution of serum micronutrient markers across age and sex.

| Age and Sex | 2.5^th^ percentile of the standard | | | |
| --- | --- | --- | --- | --- |
|  | Serum Retinol (µg/dL) | Serum Zinc (µg/dL) | Erythrocyte Folate (ng/mL) | Vitamin B_12_ (pg/mL) |
| Male:1-4y | 17.3  (n=1108) | 56.6  (n=1128) | 85.3  (n=1245) | 193.4  (n=1115) |
| Female:1-4y | 17.9  (n=1063) | 57.0  (n=1033) | 86.6  (n=1136) | 194.0  (n=1062) |
| Male:5-11y | 16.9  (n=2419) | 55.6  (n=2600) | 59.9  (n=2924) | 182.2  (n=2523) |
| Female:5-11y | 16.8  (n=2108) | 56.0  (n=2195) | 53.4  (n=2507) | 180.6  (n=2111) |
| Male:12-14y | 18  (n=688) | 54.7  (n=727) | 42.5  (n=832) | 156.4  (n=704) |
| Female:12-14y | 17.6  (n=521) | 53.9  (n=547) | 40.1  (n=620) | 159.6  (n=542) |
| Male:15-19y | 19.6  (n=975) | 55.3  (n=1027) | 37.9  (n=1206) | 142.1  (n=982) |
| Female:15-19y | 18.5  (n=624) | 53.2  (n=708) | 42.9  (n=750) | 146.6  (n=660) |

n is the number of healthy children in age and sex specific categories for each of the biomarker
